# Application of HPP for the Development of a Dessert Elaborated with Casein and Cocoa for a Dysphagia Diet

**DOI:** 10.3390/foods12040882

**Published:** 2023-02-18

**Authors:** Larisa Giura, Leyre Urtasun, Iciar Astiasaran, Diana Ansorena

**Affiliations:** 1 Centro de Investigación en Nutrición, Department of Nutrition, Food Science and Physiology, Faculty of Pharmacy and Nutrition, IDISNA—Instituto de Investigación Sanitaria de Navarra, Universidad de Navarra, 31008 Pamplona, Spain; 2 National Centre for Food Technology and Safety (CNTA), Crta-Na 134-km 53, 31570 San Adrian, Spain

**Keywords:** high-pressure processing, swallowing difficulties, texture, healthy foods, protein

## Abstract

In this study, the application of high-pressure processing (HPP) for optimizing the texture of a cocoa dessert rich in casein and developed for people with dysphagia was investigated. Different treatments (250 MPa/15 min; 600 MPa/5 min) and protein concentrations (10–15%) were combined and evaluated for choosing the optimum combination leading to an adequate texture. The selected formulation was a dessert containing 4% cocoa and 10% casein and subjected to 600 MPa for 5 min. It showed a high nutritional value (11.5% protein) and high antioxidant capacity, which was slightly affected by the HPP processing. The rheological and textural properties showed that HPP had a clear effect on the dessert structure. The loss tangent decreased from 2.692 to 0.165, indicating the transition from a liquid to a gel-like structure, which is in a suitable range for dysphagia foods. During storage (14 and 28 days at 4 °C), progressive significant changes in the structure of the dessert were observed. A decrease in all rheological and textural parameters occurred, except for the loss of tangent, which increased its value. In any case, at 28 days of storage, samples maintained the weak gel-like structure (0.686 loss tangent) that is acceptable for dysphagia management.

## 1. Introduction

It is frequently the case, especially among the elderly, that people with dysphagia suffer from undernutrition, and protein supplements are commonly needed to improve their nutritional status. Moreover, people with dysphagia usually demand a wider variety of foodstuffs in their diet with different aromas and tastes that could favor their appetite [1,2]. 

There is a consensus about the superior functional and nutritional role of milk proteins, especially when they are compared to vegetal proteins, that make them commonly used in the development of various clinical, food and pharmaceutical products [3,4]. On the other hand, the supply of antioxidant compounds could be considered a good nutritional strategy in order to increase the protection of the immune system and other metabolic pathways [5]. 

Due to its content of more than 350 different components, cocoa is considered a complex food that has good health effects [6,7]. Some of the most relevant health benefits of cocoa consumption are the antioxidant and anti-inflammatory effects, along with the modulation of the microbiota composition by acting as a probiotic agent once the phenolic compounds reach the intestine [8]. Its good antioxidant capacity is due to its composition of phenolic compounds, mainly catechins (epicatechin, epigallocatechin, gallocatechin and catechin), as well as other flavonoids such as procyanidins, anthocyanins, flavonones and flavonol glycosides [8,9,10]. All these properties make cocoa an ingredient with a high nutritional value; it is used worldwide for the preparation of various food products.

Despite the fact that the use of hydrocolloids is the main strategy for the development of dysphagia foods, in the past few years, other technologies like high-pressure processing (HPP), microwave or enzymatic treatments have been studied with the purpose of obtaining suitable textures for people with swallowing difficulties [11,12,13,14,15].

HPP technology has been used for many years as a preservation method for foods and beverages with the purpose to enlarge their shelf life. Since it is a non-thermal process, it has the advantage of preserving the nutritional and organoleptic properties of the food products by using only water and pressure instead of heat. In protein systems, HPP has the ability to disrupt noncovalent hydrophobic and electrostatic interactions and hydrogen bonds, along with a reduction in molecular volume, without affecting covalent bonds. Since the native conformation of the protein molecule is maintained, HPP provides opportunities to modify structure (agglomeration, aggregation and gel network formation) and interactions within or between food biopolymers [16]. This is why this technology has been tested for modifying texture in dairy products; it is for the sake of following the current “clean label” trend [17,18].

In the particular case of caseins, structures from sol to gel in model systems can be obtained depending on the amount of protein used and pressure applied [19]. In fact, the gel obtained by HPP at high protein concentrations is formed by the disruption of casein micelles due to the weakening of hydrophobic interaction and solubilization of calcium with HPP, followed by the re-aggregation of micelle substructures and network formation once the pressure is released [20]. In general, the research on changes in milk proteins caused by pressure has been performed at naturally occurring, low protein, concentrations, but HPP may facilitate the creation of unique structures and functionalities in high-concentration protein systems [21]. Wang and Moraru [20] pointed out that the potential of HPP to create a variety of structures using milk protein concentrates creates a new path from dairy protein sources to innovative, gel-based, high-protein foods.

In previous works performed by our group, vegetable purees and strawberry desserts enriched in proteins were designed for people with dysphagia using hydrocolloids [22,23]. On this occasion, a protein-enriched cocoa dessert to be consumed at refrigeration temperature and with an adequate texture for dysphagia management was developed by means of HPP treatment. The evolution of rheological, textural and color properties during their shelf life was assessed. 

## 2. Materials and Methods

### 2.1. Ingredients

Defatted cocoa powder (100% cocoa, Chocolates Valor, Alicante, Spain) and sugar were acquired from local supermarkets (Calahorra, La Rioja, Spain), and the micellar casein (MC) (protein content: 82 g/100 g) was acquired from the MYPROTEIN store (Manchester, UK).

### 2.2. Experimental Design

First, a series of trials with protein solutions were carried out in order to select the optimum HPP treatment to be applied (600 MPa/5 min, 250 MPa/15 min). Different MC solutions with the corresponding protein concentration (10, 12 and 15%) were prepared for these model solutions. Once the optimal % of protein and HPP treatment was chosen, the dessert samples with cocoa were elaborated.

#### 2.2.1. Protein Solutions Preparation

Solutions of micellar casein at different protein concentrations (10, 12 and 15%) were prepared by dissolving the corresponding amount of MC in water. These protein concentrations were selected based on the bibliography [20,21]. Samples were packed in 250 mL polypropylene transparent (PP) plastic bags. For each protein concentration, three different batches were prepared on the same day for each of the treatments (the control and the two HPP treatments). All samples were maintained at 4 °C prior to the analysis (approx. 24 h). 

#### 2.2.2. Dessert Elaboration 

The formulation of the dessert was: 5% cocoa, 2.5% sugar, 10% protein and 82.5% water. It was prepared as follows: Firstly, the MC was mixed with water at speed 7 (over 4400 rpm) for 5 min using a Thermomix. Then, the cocoa and the sugar were incorporated and also mixed for 5 min at speed 7 (4400 rpm) in order to achieve uniform dispersion. 

The 250 mL samples were individually packed in polypropylene transparent (PP) plastic bags. Half of the packed samples were submitted to HPP treatment, and the other half were used as controls. All of them were stored at 4 °C. The analyses were carried out after 24 h (time 0), and also after 14 and 28 days of storage in the same conditions as for the case of treated samples. Three different batches were prepared for each type of product. 

#### 2.2.3. HPP Treatment 

Two different HPP conditions were chosen based on the bibliography and applied to casein solutions using a 10 L HPP unit (Idus HPP Systems S.L.U., Noain, Navarra, Spain): 

(a) 600 MPa for 5 min or (b) 250 MPa for 15 min [19,24,25]. Results obtained with the protein model solutions helped to decide the treatment to be applied to the cocoa dessert: 600 MPa for 5 min.

### 2.3. IDDSI Measurements

The IDDSI Methods (International Dysphagia Diet Standardization Initiative) were conducted at 8 °C. The levels of the samples were characterized by the syringe test (levels 0–4 for drinks) and spoon and metal fork tests (levels 3–7 for foods) [26].

### 2.4. Nutritional Composition and Antioxidant Properties of the Cocoa Dessert

#### 2.4.1. Nutritional Composition

The nutritional composition of the dessert was determined conforming to Regulation 1169/2011 [27]. The Kjeldhal method [28] was used to determine the protein and the Soxhlet extraction method [29] was used to analyze fat. Ash and fiber were determined by the gravimetric method [30] and [31], respectively. Moisture content was determined by microwave drying [32], and carbohydrates were calculated by difference. 

#### 2.4.2. Extract Preparation for Antioxidant Properties

A freeze-dryer-cryodo (Cryodos, Telstar Industrial S.L. Terrassa, Spain) was used to lyophilize the samples. Then, 2.5 g of each lyophilized sample was weighed and added to 50 mL of ethanol (70%).

#### 2.4.3. Total Polyphenol Content

To determine the total phenolic content the Folin–Ciocalteu method was used [33]. The results were expressed as mg of gallic acid equivalents per 100 g of dessert (mg GAE/100 g). All measurements were made in quadruplicate for each dilution of the solution.

#### 2.4.4. ABTS Radical Scavenging Activity

To determine the ABTS radical Scavenging Activity, the procedure described by de Ciriano et al. [34] was used. The working solution was obtained by mixing an aqueous solution of ABTS with K_2_S_2_O_4_ (140 mM) in order to reach the final concentration of ABTS (2.45 mM). Then the mixture was kept in the dark for 12–16 h at room temperature. Before use, the ABTS working solution was diluted with ethanol 50% in order to obtain an absorbance of 0.70 ± 0.02 at 741 nm. 

For the sample measurements, 18 µL of each sample extract and 182 µL of ABTS working solution (Sigma-Aldrich Química S.A., Madrid, Spain) were allowed to react for 6 min in the dark at room temperature. After that, the absorbance was then measured at 741 nm using a spectrophotometer (FLUOStar Omega spectrofluorometric analyzer, BMG Labtechnologies, Offenburg, Germany). The decrease in absorbance was recorded as a percent of inhibition (% I) and was calculated conforming to the formula:

$$\%I=\frac{{\mathrm{Abs}}_{\mathrm{control}}-{\mathrm{Abs}}_{\mathrm{sample}}}{{\mathrm{Abs}}_{\mathrm{control}}}*100\;$$

where Abs_control_ and Abs_sample_ was the absorbance of the control and the sample, respectively, after 6 min of reaction; the final results were presented as µg Trolox/100 g of dessert and calculated using a Trolox calibration curve. All measurements were made in quadruplicate for each dilution solution.

#### 2.4.5. DPPH Radical Scavenging Activity 

The 2,2-diphenyl-1-picrylhydrazyl (DPPH) assay was determined according to the method described by Blois [35] and described in detail by Giura et al. [22]. The final results were expressed as µg Trolox/100 g of dessert and calculated using a Trolox calibration curve. All measurements were made in quadruplicate for each dilution solution.

### 2.5. Rheological Properties

A discovery HR-1 Hybrid Rheometer (TA Instruments Ltd., New Castle, DW, USA) was used in order to perform the rheological measurements. A serrated plate geometry with a 40 mm diameter was used to avoid slippage in the samples, and the measurements were conducted at 8 °C at a 3 mm gap. Following loading, the samples were released for 600 s to allow the temperature to stabilize and to recuperate from the stress caused by the geometry lowering. All measurements were made in triplicate.

#### 2.5.1. Viscosity Properties

The flow properties of protein solutions and the dessert control sample were measured by performing a flow sweep test, and the apparent viscosity of the 50 s^−1^ shear rate was recorded [36].

#### 2.5.2. Viscoelastic Properties

The linear viscoelastic region (LVR) of the dessert samples was determined by performing amplitude sweep tests, where the applied strains ranged from 0.01% to 400% and the frequency was 1 Hz, following the method used by Giura et al. [22] with some modifications. The following parameters were selected for the characterization of LVR: the storage modulus (G′) curves, the yield strain_LVR_ (%) and the corresponding yield stress_LVR_ (Pa). The limit of the LVR was taken as the value at which the G′ value deviated 5% from the plateau value, conforming to the standards ISO 6721-10 and EN/DIN EN 14,770 [37]. The yield strain_LVR_ and the yield stress_LVR_ were taken as the values of the shear strain and shear stress, respectively, at the limit of the LVE region. The flow point (Pa) was also recorded and taken as the value of the crossover point where G′ = G″. 

Frequency sweep tests were performed from 0.1 to 10 Hz at a constant strain within the LVR (obtained for each sample from the amplitude sweep tests), following the method used by Espert et al. [38] with minor modifications. The storage modulus (G′, Pa), loss modulus (G″, Pa), complex viscosity and loss tangent (tan δ, G″/G′, dimensionless) were obtained.

### 2.6. Textural Properties 

A texture analyzer (TA.XT2i Plus Texture Analyzer, Stable Micro Systems, Texture Technologies Corporation, Scarsdale, NY, USA) was used to assess the textural properties of the dessert.

A back extrusion test was conducted following the method used by Syahariza and Yong [39]. Each sample was loaded into a 60 mm diameter container which was filled 75% from its height, and a back-extrusion disc (A/BE40) of 40 mm diameter was plunged into the sample. The test settings used were 1mm/s test speed, 1 mm/s pre-speed, 10 mm/s post-speed, distance 20 mm and return distance 85 mm where the firmness (N), the consistency (N.s), the cohesiveness (N) and the viscosity index (N.s) were recorded. All measurements were made at 8 °C in triplicate. 

### 2.7. Color Measurements

The color of the dessert samples was measured with a colorimeter (CR-5, Konica Minolta Sensing Inc., Tokyo, Japan) with D65 as an illuminant and a 10 ° observer angle as a reference system. The results were expressed using CIELAB parameters (L*, a*, and b*). 

Hue angle (h) represents color shade with high values representing a more red-orange color and low values a more red-bluish color. Chroma (c) indicates a transition from grey, representing low values, to pure color, representing high values. The absolute color difference of the samples after processing and storage times was calculated as follows:

$$\Delta E=\sqrt{\left(L{*}_{0}-\;L*\right)²+\left(a{*}_{0}-\;a*\right)²+\left(b{*}_{0}-\;b*\right)²}$$

where L_0_*, a_0_* and b_0_* were the values for the control samples. A total color change (ΔE) indicates the intensity of color difference between processed and unprocessed samples and sampling times. Differences in ΔE can be classified as follows: 0–0.5, not noticeable difference; 0.5–1.5, slightly noticeable difference; 1.5–3.0, noticeable difference; 3.0–6.0, well visible difference; 6.0–12.0, great noticeable difference [40]. All the measurements were made in triplicate from different locations of the same sample.

### 2.8. Microbiological Analysis 

Microbiological analyses were performed for the control dessert at time 0 and for the treated dessert at time 0 and after 14 and 28 days of storage at 4 °C. The analyses were carried out according to microbiological-certified methods. For the mesophilic anaerobes and aerobes, the suspensions were plated on liver agar and incubated at 37 °C for 48 h and at 30 °C for 72 h, respectively [41]. For the detection of *Escherichia coli* a TBX agar was used, and the plates were incubated at 44 °C for 22 h. *Salmonella* spp. and *Listeria monocytogenes* were analyzed by VIDAS immunoanalyzer. For *Salmonella* spp. samples were first plated with buffered peptone water and incubated at 41.5 °C for 18–24 h, and for *Listeria monocytogenes* samples were plated with LMX broth incubated at 37 °C for 26–30 h [42]. All measurements were made in triplicate. 

### 2.9. Statistical Analysis 

Statistical data analysis was performed by using the STATA 15 program (Stata Corp LLC., College Station, TX, USA). The mean data of three replicates ± standard deviation were used to represent the results. Differences between treated and untreated samples were identified using a paired t-Student test (*p* ≤ 0.05). One-way analysis of variance (ANOVA) was performed to evaluate statistical significance (*p* ≤ 0.05) among all formulations followed by a post hoc test Tukey to detect statistically significant differences between samples with different sampling times. The correlation coefficient between sampling time and every texture parameter was also calculated.

## 3. Results and Discussion 

### 3.1. Selection of the Optimum HPP Treatment

HPP treatment was expected to mainly affect the protein macromolecules of the product. In this previous experiment, the aim was to attain a high level of protein concentration so that the cocoa desserts that would be further developed could be considered a significant dietary protein source for dysphagia patients. The effect of HPP on micellar caseins of milk has been widely studied [25,43,44], showing, among other effects, disruption of casein micelles as a result of colloidal calcium phosphate solubilization [43]. These changes have been shown to improve the rheological properties of fermented milk [45,46]. Moreover, other works point out that non-thermal treatments, including HPP, could enhance the formation of bioactive peptides in protein-containing foods after the digestion process, giving rise to greater potential health benefits [47].

The values of apparent viscosity at 50 s^−1^ and the results of IDDSI Testing Methods for the control protein solutions and the HPP-treated ones are presented in Table S2 and Figure S1 (Supplementary Material). IDDSI level 4 (pureed foods for solids and extremely thick for liquids) is a common target texture in dysphagia-modified foods [11,48,49]. This level does not require biting or chewing; it requires low propulsion effort and does not form oral or pharyngeal residues, which is adequate for a wide range of patients. It was chosen as the target for the developed samples.

Control samples showed IDDSI levels 2–3 and apparent viscosities of 67.7 (67.7 ± 0.3), 192.1 (192.1 ± 0.4), and 1203.0 (1203.0 ± 98.8) mPa.s for protein concentrations of 10, 12 and 15%, respectively. When HPP treatment of 250 MPa/15 min was applied, only noticeable changes were observed in the case of 12% and especially 15% protein solutions, reaching in the last case 5 level of IDDSI test. These last samples showed a gel consistency (solid-like structure) which made flow experiments and, in consequence, the measure of apparent viscosity not possible. With the HPP treatment of 600 MPa/5 min, a gel consistency was also reached in every case, making the measuring of apparent viscosity impossible. Samples with 12 and 15% of protein showed level six and samples with 10% protein, level four. 

Since only the sample containing 10% of protein and subjected to 600 MPa for 5 min reached our target texture for dysphagia foods (IDDSI 4), this protein content and the HPP treatment combination were selected to be applied in the elaboration of the cocoa dessert and submitted to the rest of analysis. This treatment also had the advantage of obtaining a certain degree of microbial inactivation, as it was used by the industry as a preservation technology to increase the self-life of foods. 

### 3.2. Nutritional Composition and Antioxidant Properties of the Cocoa Dessert

The obtained dessert showed a very interesting nutritional value with a high protein amount (11.5 ± 0.5%), a significant amount of fiber (2.6 ± 0.5%), a low amount of carbohydrates (4.2%, from which 3.2 ± 0.3% was sugar) and a very low amount of fat (0.7% ± 0.1) (Table S1, Supplementary Material). As has been stated previously, the intake of foodstuffs with high levels of high-quality proteins, as is the case with meat, is of great interest in some vulnerable subjects suffering from dysphagia [50,51]. In fact, the protein content of the developed product met the criteria for the nutrition claim “high protein content”, according to the regulation 1924/2006 [52]. A total energy of 62% of the dessert was supplied by protein, being mainly an animal-origin protein (casein) with a high biological value, supplying every essential amino acid.

On the other hand, the number of carbohydrates was low and was mainly supplied both by the added sugar necessary to dampen the bitterness of the cocoa and also by the carbohydrate molecules present in the isolated protein. The composition of macronutrients resulted in a total energetic value of 74 Kcal/100 g, which could be considered as moderate for a dessert.

Cocoa supplies some amounts of proteins, lipids and fiber but it is mainly considered to be a food with a high amount of antioxidant compounds. The analysis of the antioxidant capacity of samples reported values of 0.567 mg Trolox/100 g of sample for DPPH and 0.157 mg Trolox/100 g of sample for ABTS for the untreated dessert samples and 0.349 mg Trolox/100 g of sample for DPPH and 0.125 mg Trolox/100 g of sample for ABTS for the HPP-treated dessert samples. Regarding the phenolic content, samples contained 58.8 mg gallic acid/100 g of sample for the control and 51.4 mg gallic acid/100 g of sample for the treated samples. Similar HPP treatments in fruit purees did not report significant modifications in antioxidant activity [53]. The obtained results showed that the desserts submitted to HPP did not show relevant differences in the antioxidant capacity compared to the untreated products.

To our knowledge, no studies about the effect of HPP treatment on the antioxidant capacity of cocoa have been published so far. There are many studies showing that HPP treatments preserve the total antioxidant capacity and total phenolic compounds in fruits and vegetables. Dhenge et al. [54], analyzing the effect of different self-life treatments on orange juice, found that HPP (550 MPa for 90 s) did not affect the amount of TPC (total phenolic compounds) or the TAC (total Antioxidant Capacity) of fresh juice. 

Other works comparing the heating and HPP treatment applied to fruit purees [55] and carrot and tomato purees [56] in order to increase their self-life, found that HPP-treated products always showed higher antioxidant capacities when compared to thermally treated samples. In addition to the good antioxidant properties, it has been demonstrated that cocoa is also a good source of important minerals such as Mg, Fe and Zn, which are needed in human health for the formation of protein, of hemoglobin in red blood cells, for energy metabolism and for improving digestion, among other actions [57,58].

### 3.3. Microbiological Analysis

From the microbiological point of view, the HPP treatment (600 MPa/5 min) applied to the developed product allowed the reduction of the initial counts from 6.7 to <3.0 Log CFU/g in the case of the mesophilic aerobic counts and from 4.5 to <1 Log CFU/g in the case of the mesophilic anaerobic counts. The mesophilic bacterial counts increased from 3 to 4 Log CFU/g after 14 days of storage, reaching a level of 6.2 Log CFU/g after 28 days. On the other hand, anaerobic counts were maintained <1Log CFU/g after 14 days of storage but increased up to 5.6 Log CFU/g after 28 days. 

In case of the specific bacterial groups *L. monocytogenes* and *Salmonella* spp. were not detected, neither before nor after HPP treatment, whereas *E. coli* was detected before HPP treatment at a level of 80 CFU/g, but not after the application of HPP. These results confirm that the use of HPP technology contributes to improving the bacterial quality of the product, reducing the potential health risks associated. 

### 3.4. Rheological Properties

Before performing rheological analyses in the treated cocoa dessert, IDDSI measurements were made in order to confirm that level four was also achieved in this sample (See Figure S2, Supplementary Material).

Rheological measures were carried out at time 0 and, for treated samples also after 14 and 28 days of storage under refrigeration. Only measurements of viscoelastic properties were performed because the weak gel characteristics of the samples limited their flow properties. 

### 3.5. Viscoelastic Properties

#### 3.5.1. Oscillation Amplitude Sweep Tests

**Figure 1:**
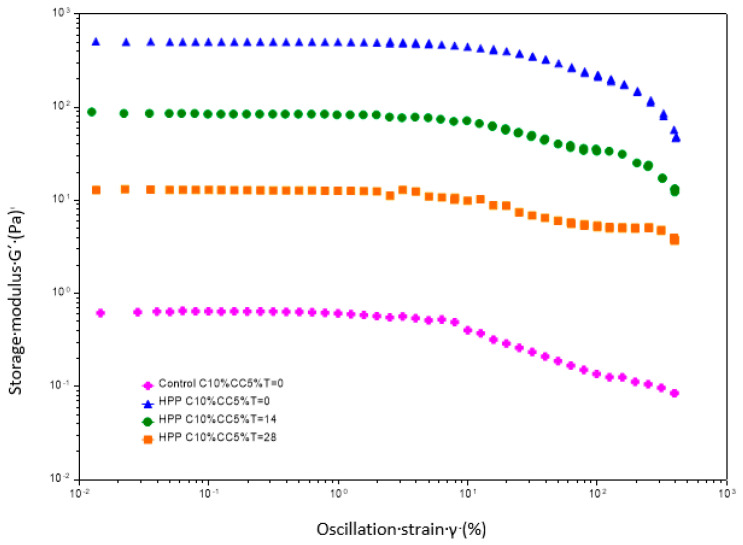
Amplitude sweep curves of storage modulus (G′) for the control sample and treated samples at different sampling times. Legend: HPP-High-pressure processing; C10%CC5%-Casein 10%, Cocoa 5%; T = 0, T = 14, T = 28. Different storage times at time 0, 14 days and 28 days.

Figure 1 shows the strain sweep for the control and the HPP-treated formulations with different storage times. Significant differences in the magnitude of the storage modulus were observed between the control and the treated samples. The highest values corresponded to HPP-treated products at time 0 during the entire strain range, pointing out higher extensional properties in the treated samples. A decrease in the intensity of the magnitude of the G′ modulus was observed as the storage time increased (14 and 28 days), indicating a change in the matrix structure during the storage period. This can be also noticed in Figure S3, Supplementary Material.

Changes in yield strain_LVR_ and yield stress_LVR_ and flow point observed during the periodical sampling are summarized in Table 1. The value of the strain and stress at the limit of LVR provides information about the stability of the material [59]. According to the increase for the G′, the high values showed by treated samples at time 0 for yield strain and yield stress indicate that HPP exerts a significant effect on the network formed by the proteins, increasing its stability and its elastic properties. In addition, a significant decrease of both parameters during storage can be appreciated, especially during the first 14 days, reaching, in the case of yield stress, values at the final of the storage period (28 days) close to those observed in the control samples.

The flow point, which is the parameter that indicates the sample transition from solid to liquid-like behavior, was not able to be determined in control samples because of its liquid-like characteristics. The highest flow point value was observed for treated samples at time 0, with decreases of 71% and 88% after 14 and 28 days of storage, respectively. 

#### 3.5.2. Oscillation Frequency Sweep Tests

**Figure 2:**
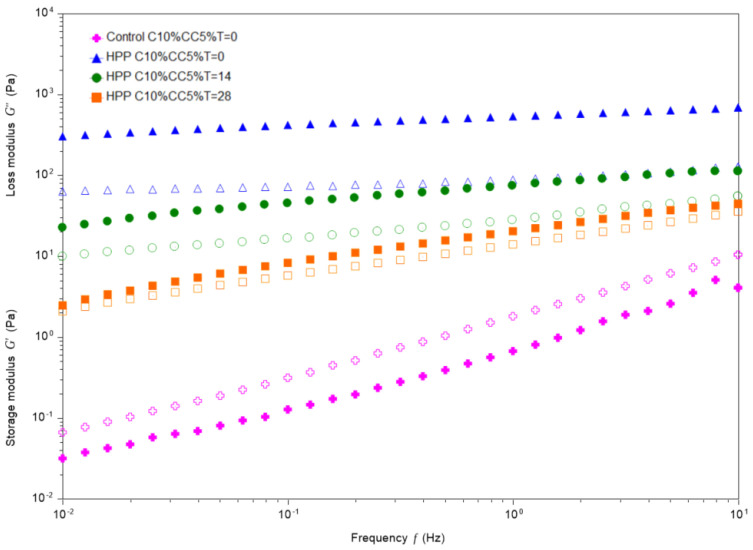
Frequency sweep curves for the control sample and treated samples at different sampling times. Storage modulus (G′): closed symbols; loss modulus (G″): open symbols. Legend: HPP-High-pressure processing; C10%CC5%-Casein 10%, Cocoa 5%; T = 0, T = 14, T = 28. Different storage times at time 0,14 days and 28 days.

Figure 2 clearly shows the effect of HPP treatment in storage and loss modulus. For control samples, G″ (loss modulus) was higher than G′ (storage modulus) during all the sweep tests, whereas for treated samples the G′ was higher than the G″, indicating the formation of a gel-like structure as a consequence of the HPP treatment. A similar effect, with an increasing trend of G′ was observed by Cadesky et al. [21] when analyzing structural changes induced by a 450 MPa HPP treatment in 10% micellar casein concentrates. This effect may be due to the network formation caused by the disruption of casein micelles, solubilization of calcium under high pressure and the re-aggregation of micelle substructures once the pressure was released [20]. Figure 2 also shows that differences between G′ and G″ were much lower in treated samples as the time of storage increased. These facts could be clearly verified in Table 2, which gathered data corresponding to the measures of G′and G″ at 1Hz of frequency. G′ is considered an indicator of the material stiffness, namely, the higher the G′ value, the stiffer the material [22]. Ratio G″/G′ (tan δ) was 18 times lower for treated samples at time 0 compared with the control samples (see Figure S4, Supplementary Material). During storage, the ratio increased with time, although the value at 28 days (0.686 ± 0.039) can be considered as a safe swallow range for dysphagia foods [60]. In addition, the complex viscosity significantly decreased (from 85.9± 7 to 3.9± 0.4) over the storage time, which reinforces the results of the rest of the parameters indicating that the dessert became more liquid with time.

### 3.6. Textural Properties

As expected, HPP significantly increased textural parameters in treated samples as compared to untreated ones. The back extrusion test (Table 3) showed that firmness, consistency, cohesiveness and index of viscosity were higher for the HPP-treated samples at time 0, compared to the control samples. 

In relation to cohesiveness, there are many works in which this parameter has been correlated with the extensional properties of the food matrix [61,62,63,64]. The extensional properties of thickened liquids for people with dysphagia have been emphasized in the last years because when the bolus is transferred to the pharyngeal phase undergo extensional deformation as a result of the dimension change compared to the oral cavity [65]. 

During the shelf life, a significant decrease in all markers was noticed, probably as a consequence of the unstable non-covalent bonds and hydrophobic interactions formed after the HPP treatment. More precisely, a linear relationship was noticed between storage time and all markers (R^2^ = 0.958, firmness; R^2^ = 0.986, consistency; R^2^ = 0.942, cohesiveness; R^2^ = 0.992, index of viscosity), with the greatest decrease for consistency pointing out a clear time-dependence of the texture properties. This finding was also reported by Wang and Moraru [66] when measuring hardness during the storage of milk protein concentrate treated with HPP. 

### 3.7. Color Measurements

Regarding color, a statistically significant although slight decrease of L*, a* and b* parameters was observed in HPP-treated samples as compared with the control samples (Table 4). The slight decrease of L*values after processing indicates that the dessert became darker, which might be due to the disintegration by the pressure of casein micelles into small fragments that increased the translucence of the dessert [67]. When HPP-treated foods are compared with thermal treated ones, better sensory scores are obtained, including those for color and appearance, although it is not always clear that there is no effect at all [44,68]. In chroma, some oscillations were observed during storage with similar values at 28 days of storage to those of control samples. Hue angle did not show differences between control and treated samples at time 0, nor during the storage of treated samples. Regarding total color through the ΔE calculation, perceptible changes could be appreciated between control and treated samples at time 0 (ΔE = 2.39). At 28 days of storage, these differences were lower (ΔE = 1.49). 

## 4. Conclusions

Protein solutions prepared with 10% of protein (casein) and subjected to HPP treatments of 600 MPa/5 min gave rise to a weak gel structure, with level 4 in the IDDSI test being considered adequate for a large share of dysphagia-affected people. When these HPP conditions were applied to a cocoa casein-enriched dessert, a food with interesting nutritional properties including significant antioxidant capacity was achieved. Moreover, its rheological and textural parameters were suitable for a wide range of patients with dysphagia. Although the gel structure of this product showed significant progressive changes, it maintained a suitable texture during the 28 days of storage at 4 °C. Color properties and microbiological parameters were also guaranteed with the applied HPP treatment. The results of this study demonstrate that high-pressure processing can be a good strategy to obtain texture-modified foods for dysphagia patients. 

## Figures and Tables

**Table 1:** Viscoelastic parameters obtained from amplitude sweep tests (yield strain, yield stress and flow point).

Amplitude Sweeps						
Sampling Time (Days)	Yield Strain_LVR_ (%)		Yield Stress_LVR_ (Pa)		Flow Point (Pa)	
	Control	HPP	Control	HPP	Control	HPP
T = 0	1.382 ± 0.025	6.022 ± 0.525 ^A,^*	0.025 ± 0.003	27.921 ± 2.951 ^A,^*	0	79.5 ± 3.4 ^A,^*
HPP T = 14	^-^	4.001 ± 0.583 ^B^	^-^	3.085 ± 0.966 ^B^	^-^	22.5 ± 1.6 ^B^
HPP T = 28	^-^	4.471 ± 0.26 ^B^	^-^	0.625 ± 0.064 ^B^	^-^	9.9 ± 0.4 ^C^

Note: Data are presented as mean ± standard deviation of three replicates. Different capital letters, in the same column, indicate significant differences (*p* < 0.05) among treated samples at different sampling times based on the post hoc Tukey test. For T = 0, the (*) in the same row indicates significant differences (*p* < 0.05) between control and treated samples. No values for control during storage time were presented because it was discarded in the following days due to its microbiological instability.

**Table 2:** Viscoelastic parameters were obtained from frequency sweep tests at 1Hz (Storage modulus, loss modulus, loss tangent and complex viscosity).

Frequency Sweeps (1 Hz)								
Sampling Time (Days)	Storage Modulus (Pa) 1 Hz		Loss Modulus (Pa) 1 Hz		Tan (Delta) 1 Hz		Complex Viscosity (Pa.s) 1 Hz	
	Control	HPP	Control	HPP	Control	HPP	Control	HPP
T = 0	0.7 ± 0.1	533.6 ± 43.07 ^A,^*	1.8 ± 0.1	87.8 ± 7.8 ^A,^*	2.692 ± 0.103	0.165 ± 0.001 ^A,^*	0.3 ± 0.0	85.9 ± 7 ^A,^*
HPP T = 14	^-^	74.1 ± 18.84 ^B^	^-^	26.9 ± 2.7 ^B^	^-^	0.374 ± 0.063 ^B^	^-^	12.6 ± 2.9 ^B^
HPP T = 28	^-^	20.3 ± 2.31 ^B^	^-^	13.9 ± 0.8 ^C^	^-^	0.686 ± 0.039 ^C^	^-^	3.9 ± 0.4 ^C^

Note: Data are presented as mean ± standard deviation of three replicates. Different capital letters, in the same column, indicate significant differences (*p* < 0.05) among treated samples at different sampling times based on the post hoc Tukey test. For T = 0, the (*) in the same row, indicates significant differences (*p* < 0.05) between control and treated samples. No values for control during storage time were presented because it was discarded in the following days due to its microbiological instability.

**Table 3:** Textural parameters obtained by back extrusion test.

Sampling Time (Days)	Firmness (*N*)		Consistency (*N.sec*)		Cohesiveness (*N*)		Index of Viscosity (*N.sec*)	
	Control	HPP	Control	HPP	Control	HPP	Control	HPP
T = 0	0.19 ± 0.01	6.87 ± 0.34 ^A,^*	3.04 ± 0.06	84.67 ± 3.05 ^A,^*	0.15 ± 0.01	6.54 ± 0.27 ^A,^*	0.09 ± 0.01	6.52 ± 1.2 ^A,^*
T = 14	^-^	2.55 ± 0.11 ^B^	^-^	37.94 ± 2.79 ^B^	^-^	2.20 ± 0.21 ^B^	^-^	3.19 ± 0.33 ^B^
T = 28	^-^	0.53 ± 0.038 ^C^	^-^	6.83 ± 0.48 ^C^	^-^	0.48 ± 0.02 ^C^	^-^	0.70 ± 0.03 ^C^

Note: Data are presented as mean ± standard deviation of three replicates. Different capital letters, in the same column, indicate significant differences (*p* < 0.05) among treated samples at different sampling times based on the post hoc Tukey test. For T = 0, the (*) in the same row, indicates significant differences (*p* < 0.05) between control and treated samples. No values for control during storage time were presented because was it discarded in the following days due to its microbiological instability.

**Table 4:** Color measurements of the samples.

Sampling Time (Days)	CIEL*a*b*									
	L*		a*		b*		c*		h*	
	Control	HPP	Control	HPP	Control	HPP	Control	HPP	Control	HPP
T = 0	35.76 ± 0.49	33.53 ± 0.32 ^A,^*	7.37 ± 0.08	6.81 ± 0.14 ^A,^*	7.29 ± 0.16	6.64 ± 0.35 ^A,^*	10.37 ± 0.17	9.51 ± 0.35 ^A,^*	44.64 ± 0.32	44.29 ± 0.91 ^A^
T = 14	^-^	33.49 ± 0.04 ^A^	^-^	6.19 ± 0.05 ^B^	^-^	5.9 ± 0.08 ^B^	^-^	8.55 ± 0.09 ^B^	^-^	43.63 ± 0.18 ^A^
T = 28	^-^	35.11 ± 0.12 ^B^	^-^	7.84 ± 0.07 ^C^	^-^	7.66 ± 0.08 ^C^	^-^	10.96 ± 0.1 ^C^	^-^	44.32 ± 0.06 ^A^

Note: Data are presented as mean ± standard deviation of three replicates. Different capital letters, in the same column, indicate significant differences (*p* < 0.05) among treated samples at different sampling times based on the post hoc Tukey test. For T = 0, the (*) in the same row, indicates significant differences (*p* < 0.05) between control and treated samples. No values for control during storage time were presented because was it discarded in the following days due to its microbiological instability.

## Data Availability

The data presented in this study are available on request from the corresponding author.

## Supplementary Materials

The following supporting information can be downloaded at: https://www.mdpi.com/article/10.3390/foods12040882/s1. Figure S1: Photos of the untreated and treated micellar casein solutions at different concentrations and HPP treatments. Figure S2: IDDSI measurements of the treated casein-enriched cocoa dessert. Figure S3: Photos of the untreated and treated casein-enriched cacao dessert at different sampling times. Figure S4: Frequency sweep curves of loss tangent of control and HPP-treated casein dessert formulations at different sampling times. Table S1: Energy content and nutritional composition of 100 g of casein cocoa dessert containing casein. Table S2: IDDSI parameters and apparent viscosity at a 50 s-^1^ shear rate for the untreated and treated protein solutions.
