# Supplementary material for: Application of HPP for the Development of a Dessert Elaborated with Casein and Cocoa for a Dysphagia Diet
Source: Foods. 2023 Feb 18;12(4):882. doi: 10.3390/foods12040882 (PMC9957160; doi:10.3390/foods12040882)

SUPPLEMENTARY MATERIAL Giura et al.

Table S1. Energy content and nutritional composition of 100 g of casein cocoa dessert containing casein

**Characteristics**	**Casein** (control)
**Energy (KJ/Kcal)**	314 KJ/74 Kcal
**Protein (g)**	11.46 ± 0.5
**Fat (g)**	0.75 ± 0.1
**Carbohydrate (g)***	4.2
**Sugars (g)**	3.2 ± 0.3
**Fiber (g)**	2.6 ± 0.5
**Ash (g)**	1.2 ± 0.1

** Calculated by difference*

Table S2. IDDSI parameters and apparent viscosity at 50 s^{-1} shear rate for the untreated and treated protein solutions

Samples	*Tº (ºc)*	*Flow test (ml)*	*Fork Drip Test*		*Fork Pressure Test*			*Spoon Tilt Test*		*Spoon Pressure Test*	*IDDSI Level*	*Apparent Viscosity (mPa.s)*
			Drips slowly in dollops/strands through the slots of a fork	*Pile above the fork*	*Make a clear pattern on the surface*	*When pressed with a fork the particles can be easily separated*	*Pressure from a fork held on its side can be used to 'cut' or break apart or flake this texture into smaller pieces*	*Easily pours from spoon when tilted*	*Holds shape on spoon (plop off the spoon if the spoon is titled)*	*Pressure from a spoon held on its side can be used to 'cut' or break this texture into smaller pieces*		
**Control (C) 10%**	8	5									2	67.7 ± 0.3
**C 10% + 600 MPa/5 min**	8	NA		X	X				X		4	ND
**C 10% + 250 MPa/ 15 min**	8	6									2	103.0 ± 1.1
**Control (C) 12%**	8	6.5									2	192.1 ± 0.4
**C 12% + 600 MPa/5 min**	8	NA					X			X	6	ND
**C 10% + 250 MPa/ 15 min**	8	9	X					X			3	1310.4 ± 195.1
**Control (C) 15%**	8	9	X					X			3	1203.0 ± 98.8
**C 15% + 600 MPa/5 min**	8	NA					X			X	6	ND
**C 15% + 250 MPa/ 15 min**	8	NA		X		X			X		5	ND

C: Casein

Figure S1. Photos of the untreated and treated micellar casein solutions at different concentrations and HPP treatments

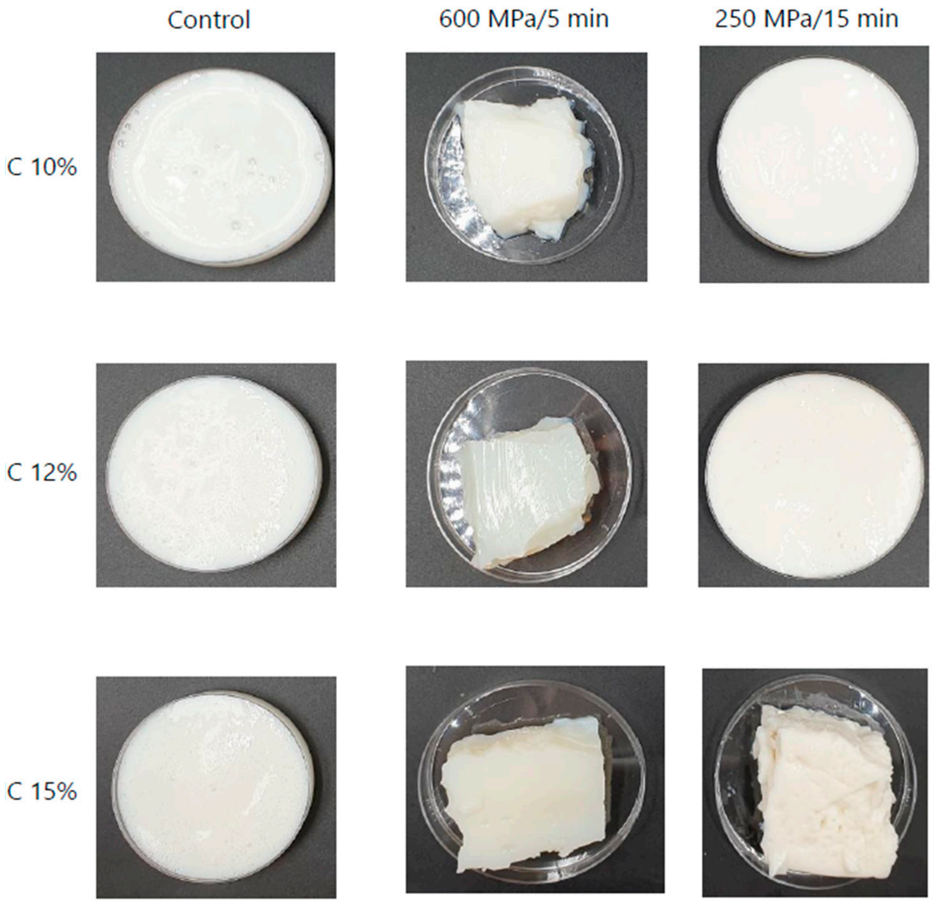

Figure S2. IDDSI measurements of the treated casein-enriched cocoa dessert

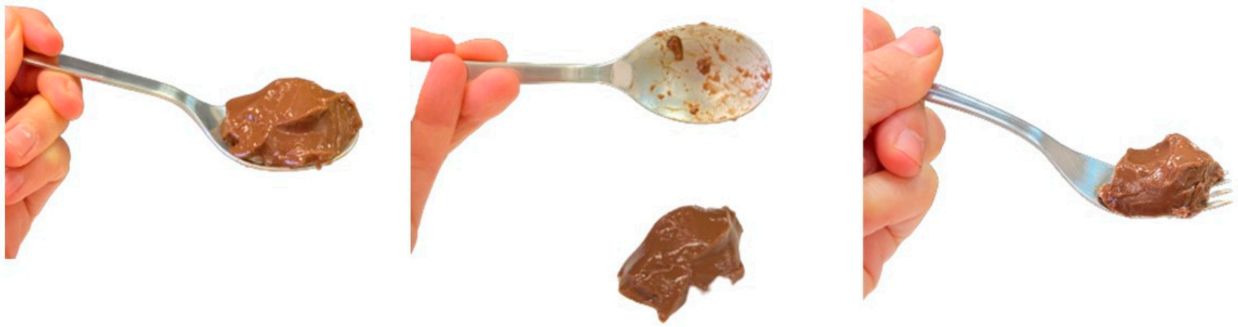

Spoon Tilt Test Spoon Tilt Test Fork Drip Test

Figure S3. Photos of the untreated and treated casein-enriched cacao dessert at different sampling times

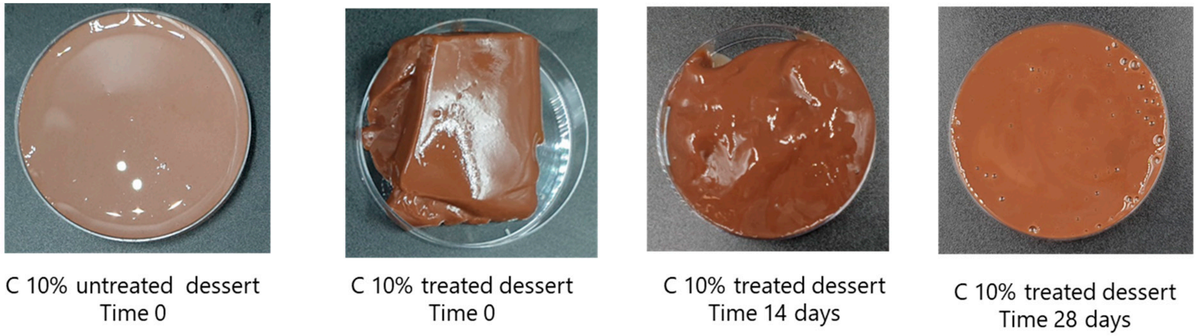

Figure S4. Frequency sweep curves of loss tangent of control and HPP treated casein dessert formulations at different sampling time

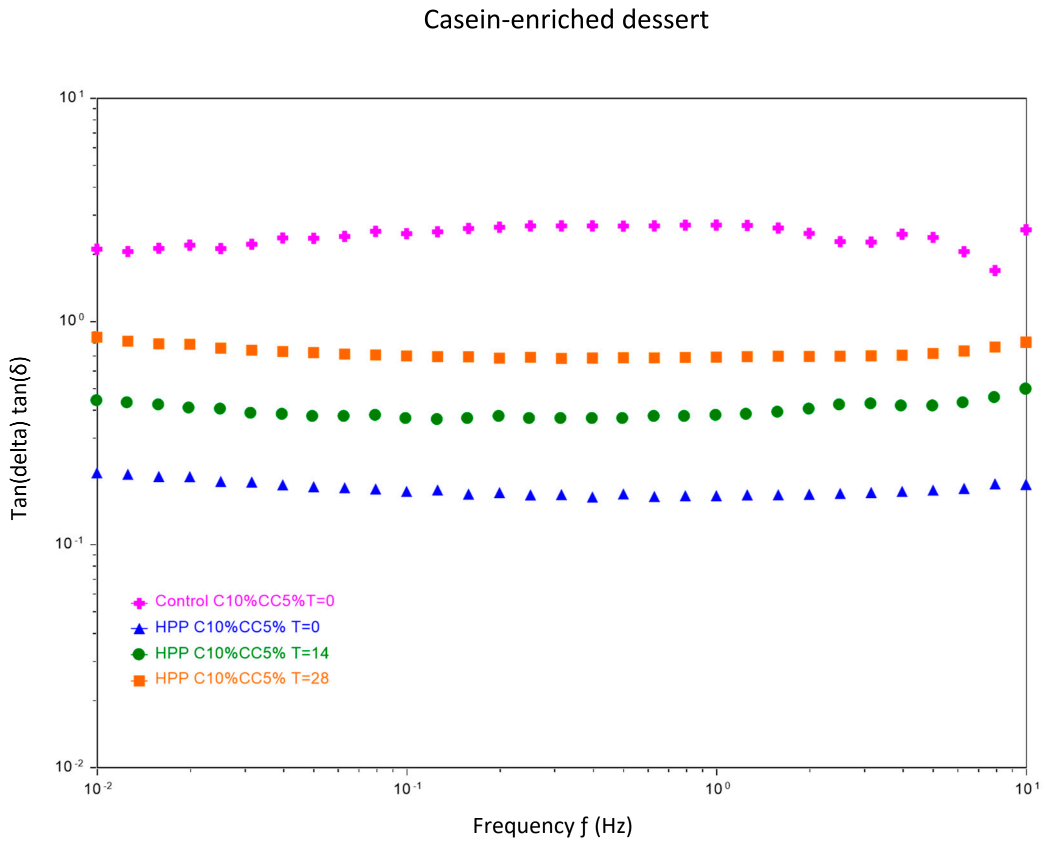

Supplement: Supplementary file 1 [file foods-12-00882-s001.zip › foods-2196917-supplementary.pdf]
